# Hepatic Cannabinoid Receptor Type 1 Mediates Alcohol-Induced Regulation of Bile Acid Enzyme Genes Expression Via CREBH

**DOI:** 10.1371/journal.pone.0068845

**Published:** 2013-07-22

**Authors:** Dipanjan Chanda, Yong-Hoon Kim, Tiangang Li, Jagannath Misra, Don-Kyu Kim, Jung Ran Kim, Joseph Kwon, Won-Il Jeong, Sung-Hoon Ahn, Tae-Sik Park, Seung-Hoi Koo, John Y L. Chiang, Chul-Ho Lee, Hueng-Sik Choi

**Affiliations:** 1 National Creative Research Initiatives Center for Nuclear Receptor Signals, Hormone Research Center, School of Biological Sciences and Technology, Chonnam National University, Gwangju, Republic of Korea; 2 Research Institute of Medical Sciences, Department of Biomedical Sciences, Chonnam National University Medical School, Gwangju, Republic of Korea; 3 Korea Research Institute of Bioscience and Biotechnology, Daejeon, Republic of Korea; 4 Department of Integrative Medical Sciences, Northeast Ohio Medical University, Rootstown, Ohio, United States of America; 5 Division of Life Sciences, College of Life Sciences and Biotechnology, Korea University, Seoul, Republic of Korea; 6 Department of Life Science, Gachon University, Sungnam, Republic of Korea; 7 Gwangju Center, Korea Basic Science Institute, Gwangju, Republic of Korea; 8 Graduate School of Medical Science and Engineering, Korea Advanced Institute of Science and Technology, Daejeon, Republic of Korea; 9 Drug Discovery Platform Technology Team, Korea Research Institute of Chemical Technology, Daejeon, Republic of Korea; Institut de Génomique Fonctionnelle de Lyon, France

## Abstract

Bile acids concentration in liver is tightly regulated to prevent cell damage. Previous studies have demonstrated that deregulation of bile acid homeostasis can lead to cholestatic liver disease. Recently, we have shown that ER-bound transcription factor *Crebh* is a downstream effector of hepatic *Cb1r* signaling pathway. In this study, we have investigated the effect of alcohol exposure on hepatic bile acid homeostasis and elucidated the mediatory roles of *Cb1r* and *Crebh* in this process. We found that alcohol exposure or *Cb1r*-agonist 2-AG treatment increases hepatic bile acid synthesis and serum ALT, AST levels *in vivo* alongwith significant increase in *Crebh* gene expression and activation. Alcohol exposure activated *Cb1r*, *Crebh,* and perturbed bile acid homeostasis. Overexpression of *Crebh* increased the expression of key bile acid synthesis enzyme genes via direct binding of Crebh to their promoters, whereas *Cb1r* knockout and *Crebh*-knockdown mice were protected against alcohol-induced perturbation of bile acid homeostasis. Interestingly, insulin treatment protected against *Cb1r*-mediated *Crebh*-induced disruption of bile acid homeostasis. Furthermore, *Crebh* expression and activation was found to be markedly increased in insulin resistance conditions and *Crebh* knockdown in diabetic mice model (*db/db*) significantly reversed alcohol-induced disruption of bile acid homeostasis. Overall, our study demonstrates a novel regulatory mechanism of hepatic bile acid metabolism by alcohol via *Cb1r*-mediated activation of *Crebh*, and suggests that targeting *Crebh* can be of therapeutic potential in ameliorating alcohol-induced perturbation of bile acid homeostasis.

## Introduction

Endogenous cannabinoids (endocannabinoids) are lipid mediators that interact with cannabinoid receptors; the two main endocannabinoids being arachidonoyl ethanolamide (AEA, anandamide) and 2-arachidonoyl glycerol (2-AG). The endocannabinoid system (ECS) includes the *Cb1r*, which has high expression levels in the brain but is also present at much lower concentrations in peripheral tissues, whereas the *Cb2r* is expressed predominantly in immune and hematopoietic cells [1]. Reports from animal studies and clinical investigations in humans have shown that in the obese state, the endocannabinoid system is hyper-activated because of impaired energy balance [2]–[4]. In obese or hyperglycemic type 2 diabetic patients, circulating levels of AEA and 2-AG are increased and elevated levels of 2-AG are found in visceral adipose tissue [2], [5], [6], while hepatic *Cb1r* activation leads to impaired insulin sensitivity as well as reduced insulin clearance in mice [7]. Mice deficient in *Cb1r* are resistant to diet-induced obesity and steatosis, and in wild type mice, chronic treatment with a *Cb1r* antagonist reversed diet-induced obesity and steatosis [8]. A recent study, using a liver-specific *Cb1r* knockout mouse model, demonstrated that peripheral *Cb1r* could be selectively targeted for the treatment of fatty liver, impaired glucose homeostasis, and dyslipidemia to reduce the neuropsychiatric side effects of nonselective *Cb1r* signaling blockade in treatment of obesity-associated conditions [9] thereby demonstrating the beneficial actions of blocking the CB1R signaling pathway to restore hepatic metabolic homeostasis.

ER stress is a state associated with perturbation of ER homeostasis and accumulation of unfolded or misfolded proteins in the ER [10]. *Crebh*, an ER-stress-activated liver enriched transcription factor, has been previously reported to transcriptionally activate acute phase response genes in the liver in response to lipopolysaccharide (LPS) and pro-inflammatory cytokines interleukin-6 (IL-6) and tumor necrosis factor α (TNFα) [11]. Recently, *Crebh* has been demonstrated to play a critical role in ER-stress-mediated regulation of iron metabolism via induction of hepcidin (*Hamp*) gene expression, in triglyceride metabolism and hepatic lipogenesis, and in the mediation of the hormonal regulation of hepatic gluconeogenesis under fasting or insulin-resistant conditions [12]–[15], thereby underlining the importance of *Crebh* in various hepatic metabolic pathways. Recent studies from our group have demonstrated that activation of *Cb1r* leads to phosphorylation of the c-Jun N-terminal Kinase (JNK) signaling pathway which in turn activates *Crebh*. This *Cb1r*-JNK-*Crebh* pathway was further demonstrated to regulate hepatic gluconeogenesis and lipid metabolism [16]–[17].

Bile acids are amphipathic detergent molecules derived from cholesterol in the liver through two pathways: the classic pathway and the alternative pathway, controlled by the rate-limiting enzymes cholesterol 7α-hydroxylase (*Cyp7a1*) and *Cyp27a1*, respectively [18]–[19]. Bile acid synthesis generates bile flow from the liver to the intestine and plays an important role in liver function, liver physiology, and metabolic regulation. As detergents, bile acids are potentially toxic, and their overall hepatic levels are tightly regulated. Thus, hepatic synthesis and the enterohepatic circulatory system work coordinately to maintain physiological bile acid homeostasis [18]–[19]. In cholestatic liver diseases, bile acids accumulate at high concentrations in the liver, resulting in hepatocyte injury, impaired liver function, fibrosis and cirrhosis [20]–[21]. A recent study has demonstrated the effect of both glucose and insulin in regulating bile acid homeostasis, with implications in diabetes and obesity [22]. Interestingly, overexpression of *Cyp7a1* in mice has been shown to regulate cholesterol homeostasis via maintenance of bile acid synthesis and secretion [23]. Previous clinical studies have demonstrated a distinct pattern of bile acids in the liver of patients with alcoholic and non-alcoholic steatohepatitis, suggesting association of specific bile acids and disease progression, possibly through bile acid-induced liver injury [20], [24]–[26].

Both ER stress and alcohol injury has been previously linked to hepatic steatosis and the correlation between bile acid levels and alcoholic hepatic steatosis is well established. Based on these premises, we examined whether bile acid metabolism is controlled by acute alcohol exposure and we investigated the contribution of *Cb1r* and *Crebh* in this context.

## Materials and Methods

### Ethics Statement

All procedures were approved by the Institutional Animal Care and Use Committee (IACUC) in Korea Research Institute of Bioscience and Biotechnology (KRIBB).

### Animal Studies

Male 8-week-old C57BL/6J mice (The Jackson Laboratory, Bar Harbor, Maine, USA), diabetic *db/db* mice (The Jackson Laboratory), and streptozotocin (STZ; 180 mg/kg; i.p., Sigma Chemical Co., St. Louis, MO, USA) treated C57BL/6J mice were used for this study. CB1 receptor knockout mice (CB1R-KO) were kindly provided by Dr. George Kunos at the National Institute on Alcohol Abuse and Alcoholism (NIAAA)/NIH, and male 8-week old CB1R-KO mice were used for the chronic alcohol study. All animals were acclimatized to a 12 hr light–dark cycle at 22±2°C with free access to food and water in a specific pathogen-free facility. To study the effect of acute alcohol injury, mice were injected with ethanol (6 mg/kg; p.o.) for 12 hrs, or pretreated with AM251 (5 mg/kg; i.p.) for 12 hrs followed by ethanol administration. For the chronic alcoholic hepatosteatosis model, mice were placed on Lieber-DeCarli liquid ethanol diet (#710260, Dyets, Bethlehem, PA) or liquid control diet (#710027, Dyets) in which alcohol was replaced isocalorically with carbohydrate. The mice were on these diets for a total of 6 wks; Ethanol was introduced gradually by increasing the content by 0.5% (*v/v*) each day or once in two days until the mice was consuming a diet containing 5% (*v/v*) ethanol. This diet was then continued for 4 more weeks. 2-AG ether (5 mg/kg; Tocris Bioscience, Bristol, UK) was administered by intraperitoneal injection in mice. Delivery of recombinant adenovirus to mice was performed via tail-vein injection. Following completion of experiments, mice were sacrificed and liver tissue and serum were collected and snap-frozen preceding bile acid analysis, total RNA isolation or protein extraction.

### Reagents and Plasmids

2-AG ether and AM251 were from Tocris Bioscience; Insulin (Norvolin R) from Green Cross (Korea). All human CYP7A1 and CYP27A1 gene promoter serial deletion luciferase constructs have been described previously [26]–[27]. pcDNA3-Flag-CREBH-N, pcDNA3-Flag-ATF6-N, Gal4DBD, Gal4DBD-CREBH-N and UAS-luciferase construct has been described previously [15]. CREBH response element mutant luciferase constructs were cloned and confirmed by DNA sequencing.

### Cell Culture and Adenoviral Infection

HepG2 hepatoma cells were maintained as described previously. Transient transfection was performed using Lipofectamine 2000 reagent (according to the manufacturer’s protocol) with treatments as indicated in the Figure Legends. For adenovirus-mediated knockdown of genes (USi and CREBHi), all further treatments with EtOH or 2-AG-ether were performed following 96 hrs of viral infection. For adenovirus-mediated overexpression of target genes (Ad-GFP, Ad-CREBH-N and Ad-ATF6-N), all further experiments were performed following 96 hrs after viral infection. For adenoviral infections, cells were washed with PBS and left for 2–3 hrs in serum-free media containing appropriate amount of viral particles (100 MOI/virus). Media was replaced with fresh growth media for an additional 72–96 hrs before any treatment. All adenoviruses used in this study have been described previously [15].

### Bile Acid Analysis

Serum and tissue bile acid analysis were performed using Bile Acid L3K Assay kit (Diagnostic Chemicals) as described previously [28].

### Analysis of Hepatic Bile Composition

Livers from mice (n = 5 per group) were processed for liquid chromatography/mass spectrometric (LC/MS) determination of bile acid composition [29], as described previously.

### Measurement of Serum ALT, AST and Total Bilirubin Levels

Mice were anesthetized with ketamine and xylazine (9∶1, 1 ml/kg, i.p.), and blood samples were collected with a heparinized capillary tube. Plasma was collected by centrifugation, aliquoted and stored at −70°C until analysis. Alanine aminotransferase (ALT), aspartate aminotransferase (AST), and total bilirubin levels were determined with an automated blood chemistry analyzer (Hitachi 7150; Tokyo, Japan).

### Isolation of Primary Hepatocytes

Primary rat hepatocytes were prepared from 200–300 g Sprague-Dawley rats by collagenase perfusion method as described previously [15]. Viability of cells was analyzed using Trypan blue staining. Cells were maintained in M199 media (Mediatech) overnight for attachment and experiments were performed as indicated. Primary human hepatocytes were obtained from the Liver Tissue and Cell Distribution System of the National Institutes of Health (S.Strom, University of Pittsburgh, PA). Hepatocytes were cultured as described previously [26]–[27].

### Quantitative PCR

Total RNA from either primary hepatocytes or liver tissue was extracted using easy spin RNA extraction kit (Intron). cDNA was generated by Superscript II enzyme (Invitrogen) and analyzed by quantitative PCR (qPCR) using a SYBR green PCR kit and Rotor Gene 6000 Real Time System (Corbett). All data were normalized to β-actin expression. All primer sequences are available upon request.

### Western Blot Analysis

Cell lysates were prepared from primary rat hepatocytes or liver tissues of experimental animals and western blot analysis was performed using mouse polyclonal CREBH [15], phospho-specific and total antibodies for JNK (Cell Signaling) and β-tubulin (Santa Cruz) antibodies as indicated.

### Chromatin Immunoprecipitation (ChIP) Assay

ChIP assay was performed according to the manufacturer’s protocol (Upstate). Briefly, HepG2 cells were transfected with reporter plasmids and treatments were performed as indicated. Cells were then fixed with 1% formaldehyde and harvested. Soluble chromatin was immunoprecipitated with polyclonal anti-CREBH antibody (Orbigen) or IgG (as a negative control). After recovering DNA, qPCR was performed using primers encompassing human CYP7A1 promoter (−300/−150) and human CYP27A1 promoter (−400/−250) region.

### Statistics

Values are expressed as mean ± SE. Statistical analysis was performed using the unpaired two-tailed Student’s t-test or ANOVA analyses. Differences were considered significant at p≤0.05.

## Results

### Alcohol Exposure and Endocannabinoid Treatment Increases Bile Acid Synthesis

**Figure 1:**
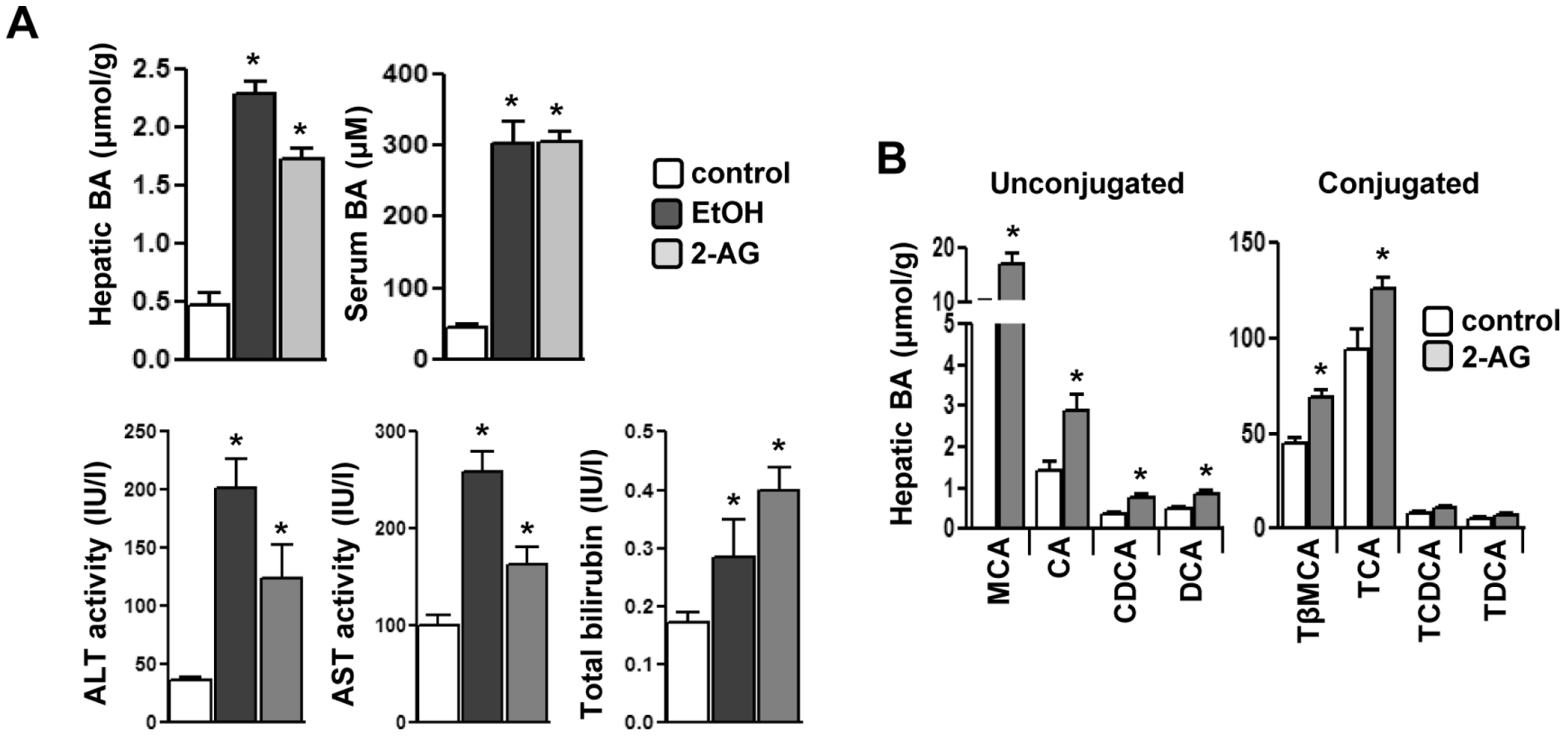
Alcohol and endocannabinoid induces hepatotoxicity and increased bile acid synthesis. (**A–B**) Mice (n = 4) were treated with EtOH or 2-AG-ether. Liver tissues and serum were obtained for total bile acid measurement, serum ALT, AST and bilirubin levels (A) and hepatic bile composition analysis (B). *p<0.01 vs. control group. Data represented as mean ± SE.

Recently, it has been reported that chronic alcohol exposure increases hepatic endocannabinoid, 2-AG levels and induces hepatic *Cb1r*, which perpetuates into a hepatic steatosis condition [30]–[31]. The acute alcohol exposure model is a much understated but equally important model to study liver injury, such as chronic alcohol exposure [32]. Therefore, we initially investigated the effect of acute alcohol exposure on bile acid synthesis and hepato-toxicity. Acute ethanol exposure or 2-AG-ether treatment significantly increased hepatic and serum bile acid levels alongwith elevated plasma levels of alanine aminotransferase (ALT), aspartate aminotransferase (AST) and bilirubin levels compared to vehicle control (Fig. 1A). Measurement of hepatic bile acid composition in 2-AG-ether treated mice revealed that the contribution of unconjugated bile acids (muricholic acid, cholic acid, chenodeoxycholic acid and deoxycholic acid) to the total bile acid pool was significantly higher compared to vehicle control or to their taurine-conjugated counterparts (Fig. 1B). These findings suggest that alcohol exposure leads to increased biosynthesis of endocannabinoid 2-AG and acute alcohol exposure or 2-AG-ether treatment leads to hepatocellular damage due to increased ALT, AST and total bilirubin levels in serum.

### Alcohol Regulates Bile acid Metabolism via *Crebh*

**Figure 2:**
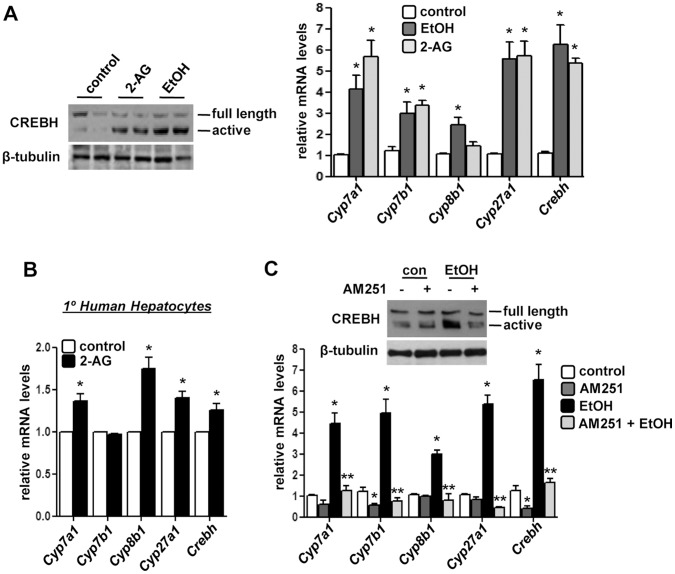
Acute alcohol and endocannabinoid regulates key bile acid genes expression. (**A–B**) Mice (n = 4) or primary human hepatocytes (n = 3) were treated with EtOH or 2-AG-ether. Liver tissues were obtained and protein and total RNA was extracted for western blot and qPCR analyses, respectively. *p<0.01 vs. control group. (**C**) Mice (n = 4) were treated with AM251 or AM251+EtOH as indicated. Liver tissues were obtained and protein and total RNA was extracted for western blot and qPCR analyses, respectively. *p<0.01 vs. control group, **p<0.01 vs. EtOH-treated group. Data represents mean ± SE.

Ethanol has been reported to promote oxidative and ER stress condition [33]. Therefore, we suspected that alcohol or 2-AG-ether treatments might elicit a stress response. Indeed, we observed that both treatments led to the cleavage and activation of *Crebh*, as observed by the generation of the 50 kDa active form of Crebh protein (Fig. 2A), indicating a correlation between stress signal and alcohol in liver. Next, we assessed the gene expression of several key bile acid biosynthetic enzymes in hepatocytes. Acute ethanol exposure or 2-AG-ether treatment significantly induced mRNA levels of *Cyp7a1*, *Cyp7b1*, *Cyp8b1* and *Cyp27a1* as well as *Crebh* in mouse livers (Fig. 2A). Similarly, 2-AG-ether treatment in primary human hepatocytes showed significant increases in *CYP7A1*, *CYP8B1*, *CYP27A1* and *CREBH* mRNA levels but surprisingly, no change was observed in *CYP7B1* expression (Fig. 2B).

Next, to reconfirm that the alcohol-mediated regulation of bile acid metabolism occurs via activation of the *Cb1r* signaling pathway, we treated mice with the *Cb1r*-antagonist AM251. Treatment of mice with AM251 dramatically attenuated the alcohol-mediated induction of bile acid biosynthetic genes, *Crebh* mRNA level, and generation of the active form of Crebh protein (Fig. 2C), thereby indicating that the alcohol-mediated regulation of bile acid metabolism occurs via the cannabinoid signaling pathway. Overall, these results suggest a mediatory role of *Crebh* in regulation of bile acid metabolism by acute alcohol exposure.

### *Crebh* is a Transcriptional Activator of Key Bile Acid Enzyme Genes

**Figure 3:**
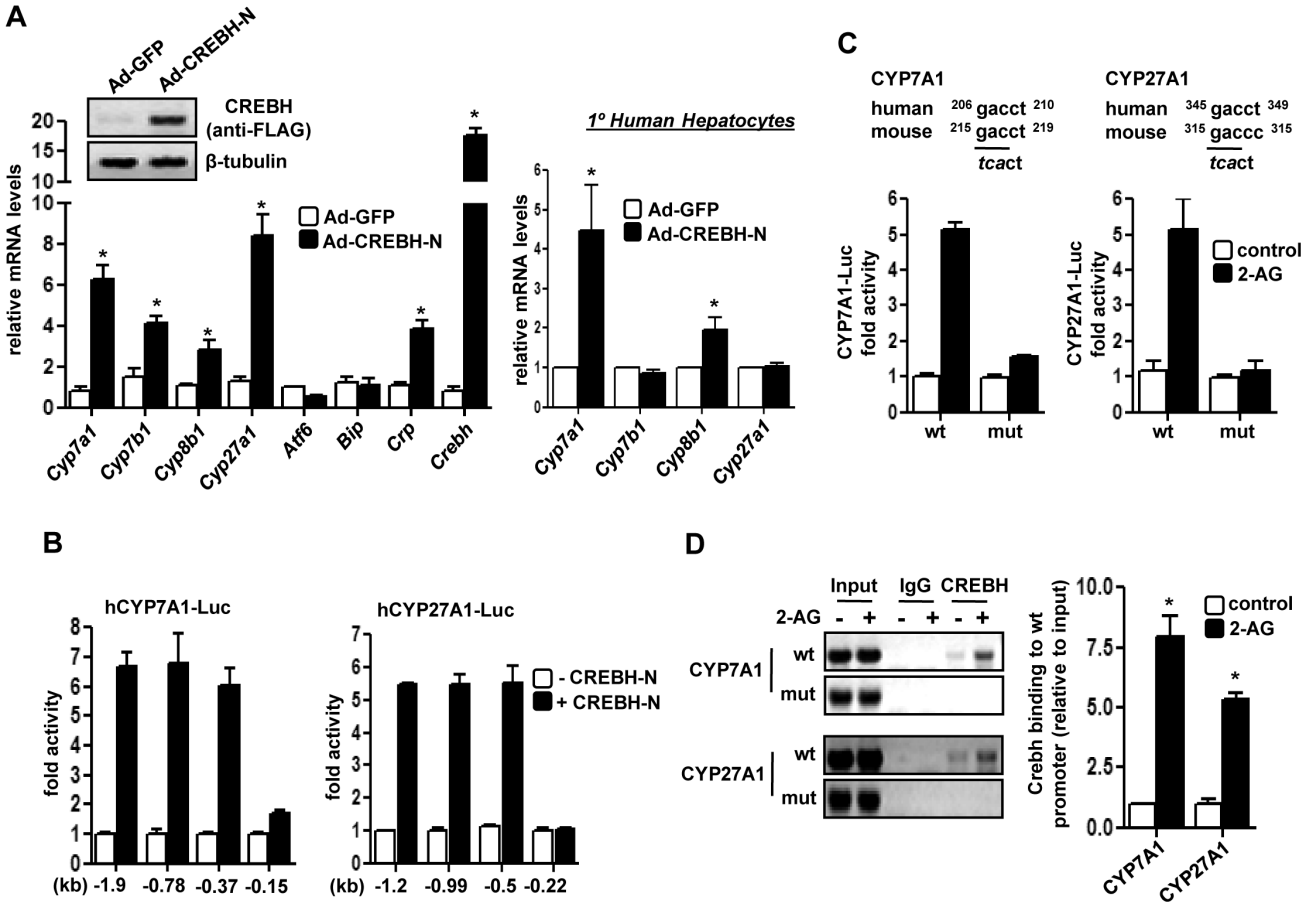
Transcriptional regulation of bile acid enzyme genes by *Crebh.* (**A**) Mice (n = 5) or primary human hepatocytes (n = 3) were infected with indicated adenoviruses for 96 hrs. Liver tissues were obtained and protein and total RNA was extracted for western blot and qPCR analyses, respectively. *p<0.05 vs. Ad-GFP group. (**B**) HepG2 cells were co-transfected with CREBH-N and different CYP7A1-Luc and CYP27A1-Luc promoter constructs, and luciferase assay was performed. (**C–D**) HepG2 cells were transfected with wild type (wt) or CREBH-mutant (mut) constructs of CYP7A1-Luc or CYP27A1-Luc followed by 2-AG-ether treatment for 12 hrs and luciferase assay was performed (D) or immunoprecipitation of HepG2 chromatin from cells exposed to DMSO (control) or 2-AG-ether was performed with IgG or Crebh antibody (E). Promoter regions were amplified by PCR, as depicted. Percentage of DNA immunoprecipitated with Crebh antibody relative to input chromatin was quantified by qPCR. *p<0.05 vs. control. Data represents mean ± SE.

To further clarify the role of *Crebh* in alcohol and endocannabinoid-mediated regulation of bile acid homeostasis *in vivo,* we intravenously injected *Crebh* adenovirus into mice. *Crebh* overexpression led to a marked increase in expression of bile acid biosynthetic genes *in vivo* (Fig. 3A) but, interestingly, had no significant effect on *CYP7B1* and *Cyp27A1* mRNA levels in primary human hepatocytes (Fig. 3A). *Atf6* and *Bip* mRNA levels showed no significant change upon *Crebh* overexpression and C-reactive protein (*Crp*), a previously reported acute-phase response gene and target of *Crebh* [9], mRNA levels were significantly increased upon *Crebh* over-expression (Fig. 3B), thereby confirming the validity of *Crebh* overexpression. Overall, these results indicate that *Crebh* plays a critical role in mediating alcohol-induced regulation of hepatic bile acid homeostasis.

Next, we investigated the mechanism for transcriptional activation of human *Cyp7a1* and *Cyp27a1* gene promoters by *Crebh*. Co-transfection assays with a vector expressing the *Crebh* N-terminal active form (CREBH-N) significantly activated both *Cyp7a1* and *Cyp27a1* gene promoters. Reporter assays of serial deletion constructs identified a putative binding site for CREBH on both gene promoters (Fig. 3B). To further confirm the role of the putative *Crebh* binding sites in driving *Cyp7a1* and *Cyp27a1* promoter activities, we performed transfection assays with reporters containing mutant *Crebh* binding sites (Fig. 3C). 2-AG-stimulated *Cyp7a1* and *Cyp27a1* promoter activity was dramatically abrogated (∼80%) in mutant promoter constructs compared to wild type. *Crebh* binding to the endogenous promoters upon 2-AG ether stimulation was confirmed by ChIP assay with a specific antibody for Crebh (Fig. 3D). Overall, these results indicate that the putative *Crebh*-responsive elements identified in both *Cyp7a1* and *Cyp27a1* promoters are responsive to 2-AG-ether treatment.

### Genetic Ablation of *Cb1r* Attenuates Alcohol-induced Perturbation of Bile Acid Homeostasis

**Figure 4:**
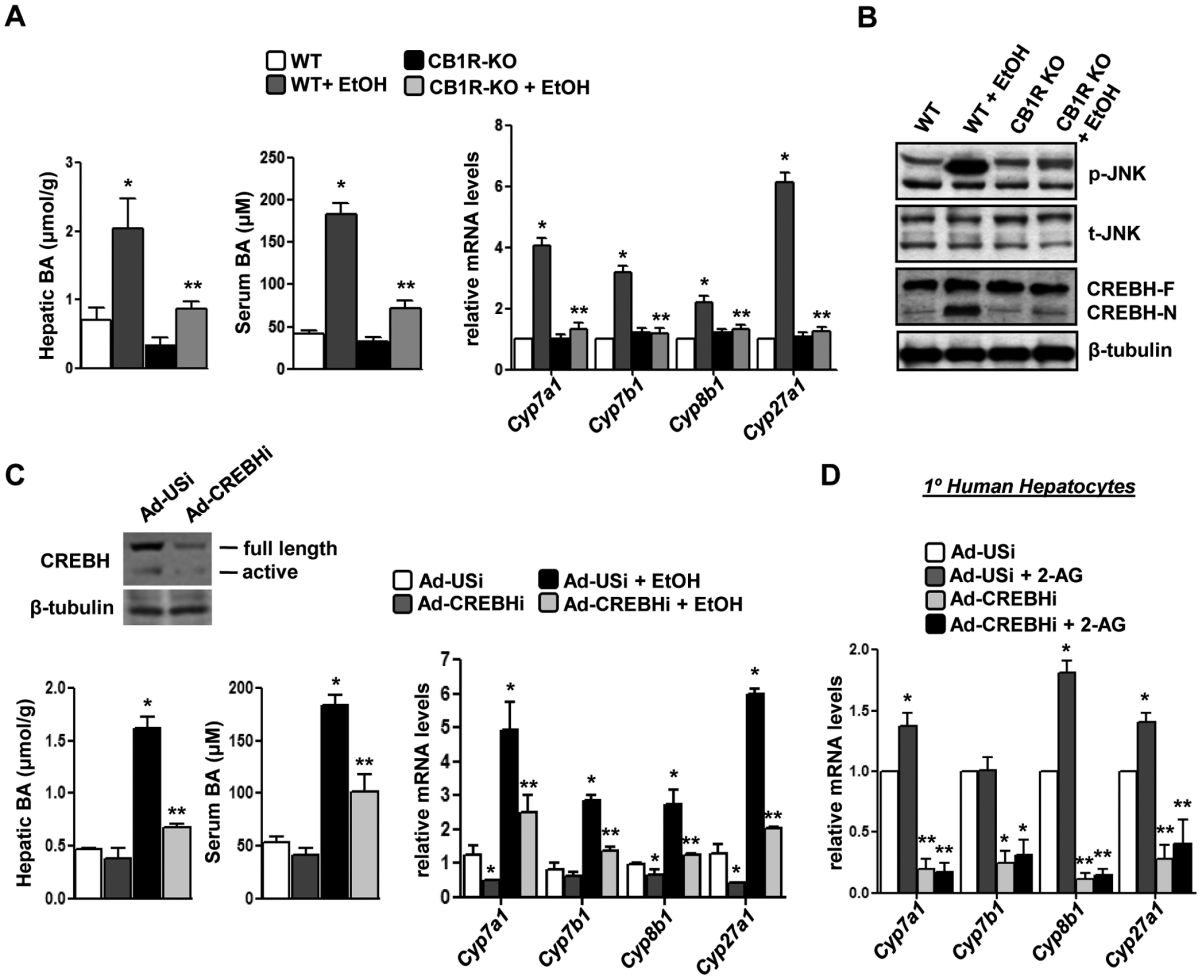
*Cb1r*-deficiency or *Crebh* knockdown reverses attenuates alcohol-mediated induction of bile acid enzyme genes. (**A–B**) Wild type (WT) or CB1R KO mice (n = 4) were treated with EtOH. Liver tissues were obtained and protein and total RNA was extracted for western blot and qPCR analyses, respectively. Liver tissues and serum were further utilized for total bile acid measurement. *p<0.05 vs. WT group, **p<0.02 vs. WT+EtOH group. (**C–D**) Mice (n = 4–5) or primary human hepatocytes (n = 3) were infected with indicated adenoviruses for 96 hrs followed by treatment with EtOH or 2-AG-ether as indicated. Liver tissues were obtained and protein and total RNA was extracted for western blot and qPCR analyses, respectively. Liver tissues and serum were further utilized for total bile acid measurement. *p<0.05 vs. Ad-USi group, **p<0.01 vs. EtOH or 2-AG-ether-treated group. Data represents mean ± SE.

Next, to confirm the role of *Cb1r* in mediating the effects of alcohol on bile acid metabolism, we challenged wild type (WT) and *Cb1r* knockout mice (CB1R-KO) with chronic exposure to ethanol. Chronic alcohol exposure led to a significant increase in mRNA levels of bile acid enzyme genes (*Cyp7a1, Cyp7b1, Cyp8a1* and *Cyp27a1*) in wild type mice liver but showed no noticeable increase in knockout mice under similar exposure (Fig. 4A). Furthermore, both hepatic and serum bile acid levels were significantly increased in wild type mice upon alcohol challenge and this increase in bile acid synthesis was abrogated in CB1R-KO (Fig. 4A), thereby suggesting the mediatory role of *Cb1r* in alcohol-induced perturbation of bile acid homeostasis. Previously, we have demonstrated that activated *Cb1r* phosphorylates JNK signaling to activate *Crebh* [16]–[17]. Therefore, we next assessed the effect of chronic alcohol on JNK phosphorylation and Crebh activation in wild type and *Cb1r* knockout mice. Alcohol exposure in wild type mice increased phosphorylation and activation of the JNK signaling pathway that led to an increase in the active form of *Crebh* (CREBH-N), whereas both JNK phosphorylation and *Crebh* activation was significantly diminished in knockout mice challenged with alcohol (Fig. 4B). These results clearly indicate that perturbation of bile acid homeostasis upon alcohol exposure is mediated by activation of *Cb1r* and its downstream effectors like phosphorylation of JNK signaling pathway and subsequent activation of *Crebh*.

### *Crebh* Deficiency Abrogates Alcohol-mediated Regulation of Bile Acid Metabolism

Next, we investigated the effect of *Crebh* knockdown by adenoviral overexpression of *Crebh* shRNA (Ad-CREBHi) on alcohol-induced deregulation of bile acid metabolism. Acute alcohol exposure led to a marked increase in bile acid biosynthetic genes expression as well as serum and hepatic bile acid levels in control shRNA (Ad-USi) infected mice but failed to appreciably induce the expression of these genes or increase serum and hepatic bile acid levels in CREBHi infected mice (Fig. 4C). Consistent with these results, 2-AG-ether-mediated induction of *CYP7A1*, *CYP8B1* or *CYP27A1* genes were significantly abrogated upon *CREBH* knockdown in primary human hepatocytes (Fig. 4D). Overall, these results clearly indicate a crucial role for *Crebh* in mediating the effects of acute alcohol and 2-AG in regulating bile acid homeostasis in mice and humans.

### Insulin Protects against Endocannabinoid-induced Perturbation of Bile Acid Homeostasis

**Figure 5:**
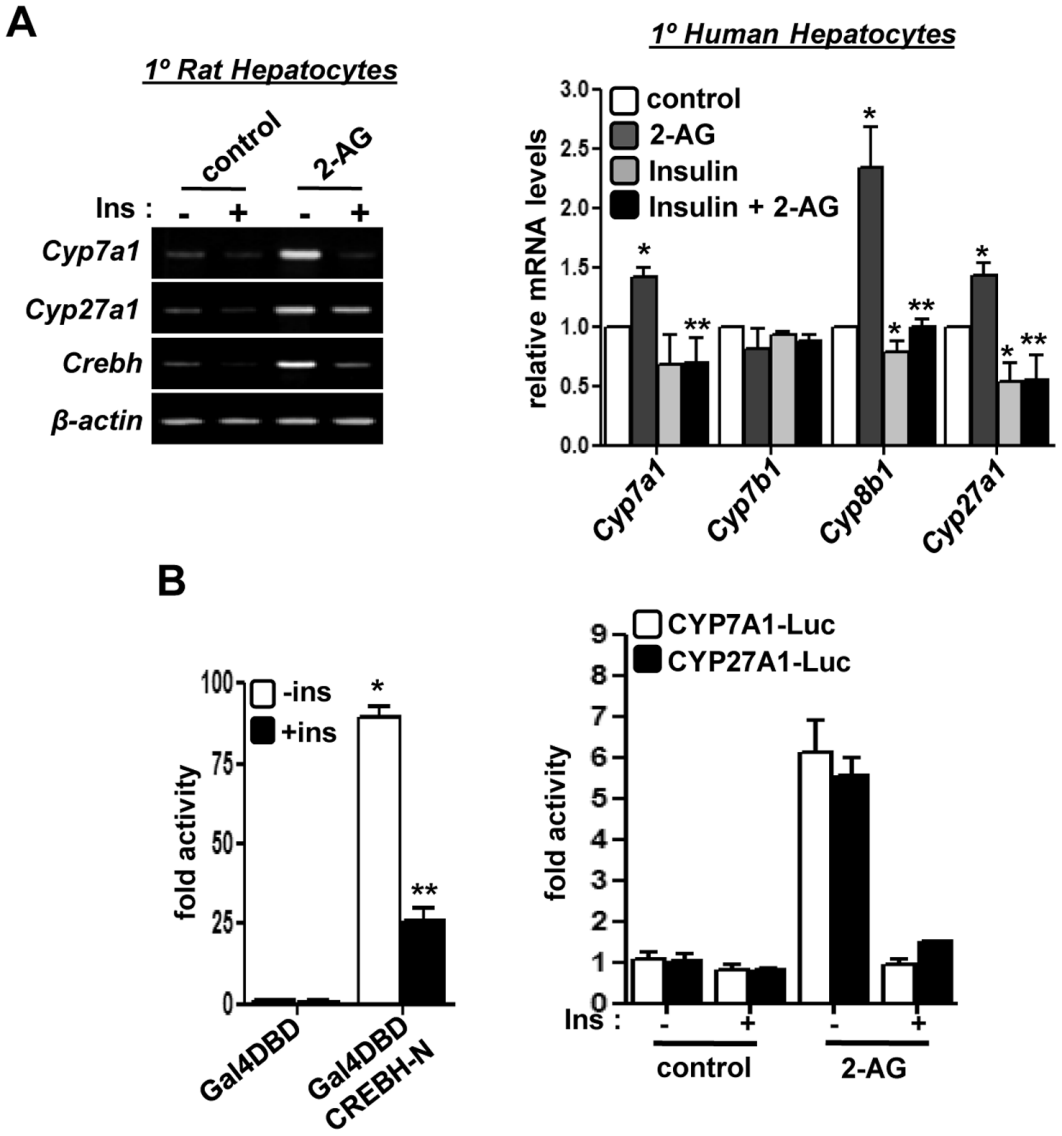
Insulin treatment protects against alcohol-induced deregulation of bile acid homeostasis. (**A**) Primary rat and human hepatocytes (n = 3) were treated with 2-AG-ether in the absence or presence of insulin as indicated. RNA was extracted for semi-quantitative PCR analysis (left) or qRT-PCR analysis (right). Images are representative of 3 independent experiments. *p<0.05 vs. control, **p<0.05 vs. 2-AG-ether-treatment. (**B**) HepG2 cells were co-transfected with Gal4-luciferase reporter and Gal4DBD or Gal4DBD CREBH-N. 36 hrs post transfection cells were treated with insulin for further 12 hrs (left). Luciferase assay was performed using CYP7A1 and CYP27A1-promoter luciferase reporters with indicated treatments (right). Luciferase activity was assessed and represented as fold activity. *p<0.05 vs. untreated control. Data represents mean ± SE.

**Figure 6:**
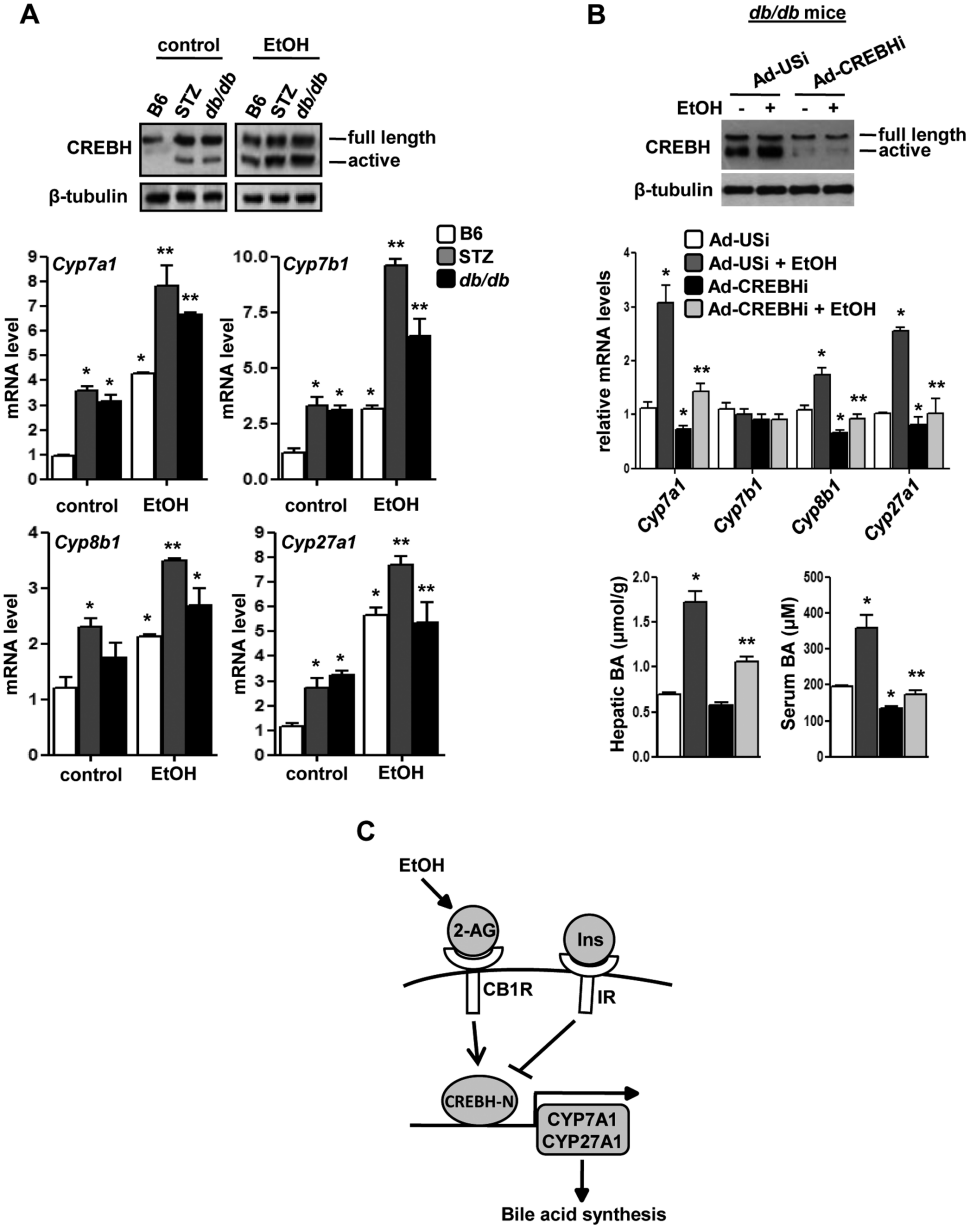
*Crebh* knockdown restores bile acid homeostasis in alcohol-exposed insulin-resistant mice model. (**A**) B6, STZ-treated and *db/db* mice (n = 4–5) were treated with EtOH as indicated. Liver tissues were obtained and protein and total RNA was extracted for western blot and qPCR analyses, respectively. *p<0.01 vs. B6 control group, **p<0.01 vs. STZ-treated and *db/db* group. (**B**) *db/db* mice (n = 5) were infected with the indicated adenoviruses for 96 hrs followed by treatment with EtOH. Liver tissues were obtained, protein and total RNA was extracted for western blot and qPCR analyses, respectively. Liver tissues and serum were further utilized for total bile acid measurement. *p<0.01 vs. Ad-USi group, **p<0.01 vs. Ad-USi+EtOH group. Data (A-B) represents mean ± SE. (**C**) Proposed model for the regulation of hepatic bile acid homeostasis by acute alcohol exposure via CB1R and CREBH. Acute alcohol exposure and endocannabinoid signaling induce *Crebh* gene expression and generates active *Crebh* (CREBH-N). Active *Crebh* regulates the expression of *Cyp7a1* and *Cyp27a1* directly and that of *Cyp7b1* and *Cyp8b1* indirectly, which leads to elevated hepatic bile acid concentrations. This, in turn, causes liver injury. On the other hand, insulin receptor signaling maintains bile acid homeostasis via inhibition of *Crebh* gene expression and activity.

Recently, we have demonstrated that insulin regulates *Crebh* gene expression [15]. Therefore, upon investigating the mechanism of insulin-mediated regulation of *Crebh* expression and the potential effect of insulin in bile acid homeostasis, we found that insulin treatment strongly inhibited 2-AG-ether-induced mRNA levels of *Cyp7a1*, *Cyp27a1* and *Crebh* in both rat and human primary hepatocytes (Fig. 5A). Furthermore, insulin inhibited the transcriptional activity of Crebh and 2-AG-ether-induced *CYP7A1* and *CYP27A1* gene promoter activity (Fig. 6B). These results indicate that under normal conditions insulin plays a crucial role in maintaining bile acid homeostasis via regulation of *Crebh* transcriptional activity.

### Alcohol-induced Perturbation of Bile Acid Homeostasis is Exacerbated in Insulin Deficient and Insulin Resistant Conditions

Previous studies have demonstrated the correlation between insulin resistance and high bile acid levels in human patients [19]–[21]. Therefore, we wondered the effect of insulin resistance on bile acid metabolism when exposed to acute alcohol injury. We initially compared the modulation of bile homeostasis by alcohol in insulin deficiency and resistance conditions using streptozotocin (STZ)-treated mice (type I diabetes model) and *db/db* mice (type II diabetes model), respectively. We observed that the active form of Crebh protein was abnormally elevated in STZ and *db/db* mice in both untreated conditions as well as alcohol treated animals in comparison to normal littermates (Fig. 6A), suggesting increased *Crebh* activity under these pathological conditions. Furthermore, expression of bile acid biosynthetic genes was also markedly increased under these conditions (Fig. 6A), indicating perturbation of bile acid homeostasis under these conditions and a possible involvement of *Crebh* in mediating the effect of alcohol on bile acid homeostasis during metabolic perturbations like insulin resistance.

Therefore, finally, we investigated the effect of *Crebh* deficiency on acute alcohol exposure in *db/db* mice. *Crebh* knockdown significantly attenuated alcohol-mediated increases in mRNA levels of *Cyp7a1*, *Cyp8b1* and *Cyp27a1* genes in *db/db* mice (Fig. 6B). However, *Cyp7b1* gene expression levels showed no significant change under these conditions. Serum and hepatic bile acid levels were significantly higher upon alcohol exposure in these mice and *Crebh* knockdown led to a considerable decrease in bile acid levels under basal conditions (serum levels) or under alcohol exposure (both serum and hepatic levels). Overall, our current findings indicate that insulin resistance of differing etiologies, along with acute alcohol injury, causes abnormal upregulation of bile acid biosynthetic genes as well as *Crebh* and suggests that high levels of *Crebh* exacerbate the effects of alcohol injury to the liver in insulin-deficient and resistant conditions.

## Discussion

The role of *Crebh* in hepatic transcriptional regulation has recently been established in studies demonstrating that *Crebh* is a crucial mediator of the acute inflammatory response elicited by various pro-inflammatory cytokines and transcriptional activation of acute-phase response genes, such as serum amyloid P component (*Srp*) and *Crp,* in the liver; *Crebh* also modulates gene expression of the iron-regulatory hormone, hepcidin, as well several key genes involved in hepatic triglyceride metabolism and lipogenesis [11]–[14]. Recently, we demonstrated that *Crebh* plays a critical role in regulating hepatic gluconeogenesis in fasting conditions as well as in diet-induced or genetically modified insulin resistance models [15]. Due to its liver-enriched expression pattern and stress-sensory activation, *Crebh* has emerged as a key player in various hepatic metabolic pathways. Bile acid metabolism is another crucial hepatic metabolic pathway and bile acids, being powerful detergents, are tightly regulated to prevent hepatotoxicity and liver injury. Our current study demonstrates that alcohol and endocannabinoids, two major factors shown by various studies to deregulate liver function that results in various metabolic syndromes, elicits a stress-response leading to activation of *Crebh* and alters expression of multiple genes involved in bile acid metabolism, thereby suggesting a link between alcohol and cannabinoid receptor signaling with ER stress and deregulated bile acid metabolism in the liver (Fig. 6C).

Previous studies have demonstrated the disruptive influence of ER stress on various metabolic pathways [33]–[37]. Using genetic ablation of ER stress-sensing pathways or ER quality control genes, it was demonstrated that ER stress mediates hepatic steatosis [38]. However, the question remained of which factors mediate the negative effects of ER stress in disrupting metabolic homeostasis. Previously, acute and/or chronic alcohol exposure has been reported to generate ER stress conditions and reports suggest that alcohol activates hepatic *Cb1r* signaling via upregulation of endocannabinoid, 2-AG, and causes alcoholic steatohepatitis by inducing lipogenic gene expression [30]. Our study demonstrates that alcohol and endocannabinoid induces *Crebh* gene expression and generates active *Crebh* via *Cb1r*. Genetic ablation of *Cb1r* or knockdown of *Cb1r* in wild type mice significantly reversed the effect of alcohol exposure on *Crebh* induction and activation as well as its downstream effects on bile acid enzyme gene expression. This adaptive response of *Crebh* activation upon acute alcohol injury influenced bile acid metabolism through activation of key bile acid biosynthetic genes, *Cyp7a1* and *Cyp27a1*, the rate-determining enzyme genes for classical and alternative pathways of bile acid synthesis from cholesterol. Crebh occupancy in these gene promoters may serve as a stress sensor for endogenous or exogenous signals perturbing bile acid homeostasis. In this context, a recent study demonstrated that genetic ablation of forkhead box protein (*Foxa2*) sensitizes mice to cholic acid diet treatment, which results in toxic accumulation of hepatic bile acids and causes ER stress and liver injury [39]. However, from our study, we conclude that it is the stress-condition that induces bile acid enzyme genes and precedes the disproportionate increase in bile acid synthesis. A plausible explanation behind this discrepancy might be a vicious cycle of stressful conditions that deregulate bile acid metabolism, as has been observed from our results. The deregulation of bile acid metabolism leads to increased accumulation of toxic bile acids which in turn further stresses the ER, demonstrated in *Foxa2*-deficient mice.

Recent studies have demonstrated the presence of an unfolded protein response (UPR) in the liver and adipose tissue of insulin-resistant rodents, and counteraction of UPR has been shown to improve the insulin resistance in these animals [40]–[41]. Liver function is also sensitive to environmental or genetic perturbation, and a number of these perturbations, including both alcoholic and nonalcoholic steatohepatitis, viral hepatitis, hyperhomocysteinemia, acute exposure to hepatotoxins, and high carbohydrate or high fat diets, have been suggested to lead to hepatic ER stress [36], [41]–[42]. Previously, it was reported that *Atf6* protects against hepatic steatosis and contributes to the overall maintenance of homeostasis in the ER during stress [38]. Consistent with the previous report, our results also suggest that *Atf6* may not be involved in aberrant upregulation of bile acid homeostasis by acute alcohol exposure. Another study demonstrated, using an intragastric ethanol feeding model, that CCAAT/enhancer-binding protein (C/EBP) homologous protein (*Chop*) knockout mice have a marked absence of hepatocellular apoptosis as well as reduced cholestasis-induced liver fibrosis by preventing hepatocyte injury [43]. Our results indicate that excessive production of bile acids along with increase in serum ALT, AST and bilirubin levels occur upon alcohol-dependent activation of *Crebh*. Therefore, it would be interesting to ascertain whether *Crebh* works concertedly with *Chop* under these stress conditions.

Insulin resistance conditions have been previously reported to exhibit increased levels of hepatic bile acids. In our previous study, we observed the abundance of the active *Crebh* form under these conditions [15]. That led us to investigate the effect of alcohol on *Crebh*-mediated regulation of bile acid homeostasis under insulin resistance conditions in this study. Consistent with previous reports [18]–[21], [25], we found that alcohol treatment markedly increased hepatic and serum bile levels (data not shown) along with a disproportionate increase in *Crebh* expression and activity in diabetic animals, which subsequently induced bile acid enzyme gene expression. Most of the studies related to effects of alcohol have been performed using a chronic alcohol injury model over an extensive period of time (six to eight weeks). However, recent reports suggest that acute alcohol injury is a more relevant model with serious deleterious effects in the liver; despite this, it is not a frequently studied model [32]. In our current model of study, we used transient knockdown of *Crebh* as well as *Cb1r* gene expression or challenged *Cb1r* knockout mice with an acute alcohol injury condition to elucidate the potential harmful effects of binge drinking in liver. Further studies with liver-specific genetic ablation of *Crebh* to delineate its contribution to acute or chronic alcohol consumption may be warranted.

In summary, our data provides a connection between alcohol and endocannabinoid signaling mediated upregulation of “stress sensory” ER-bound transcription factor *Crebh* and its role in hepatic bile acid metabolism. Under alcohol injury, the Cb1r signaling pathway gets abnormally activated leading to induction and activation of a stress-induced transcription factor, *Crebh,* which plays a significant role in up-regulating both the classical and alternative bile acid synthesis pathways. Alcohol consumption has long been associated with several metabolic complications, therefore, identification of the *Cb1r-Crebh* pathway as a critical mediator of this effect would expand our knowledge to understand the complex mechanisms involved in tightly regulating hepatic bile acid metabolism.

## References

[1] Pacher P, Bátkai S, Kunos G (2006) The endocannabinoid system as an emerging target for pharmacotherapy. Pharmacol Rev 58: 389–462. 16968947 10.1124/pr.58.3.2 PMC2241751

[2] Matias I, Gonthier MP, Orlando P, Martiadis V, De Petrocellis L, et al (2006) Regulation, function, and dysregulation of endocannabinoids in models of adipose and beta-pancreatic cells and in obesity and hyperglycemia. J Clin Endocrinol Metab 91: 3171–3180. 16684820 10.1210/jc.2005-2679

[3] Osei-Hyiaman D, DePetrillo M, Pacher P, Liu J, Radaeva S, et al (2005) Endocannabinoid activation at hepatic CB1 receptors stimulates fatty acid synthesis and contributes to diet-induced obesity. J Clin Invest 115: 1298–1305. 15864349 10.1172/JCI23057 PMC1087161

[4] Di Marzo V, Goparaju SK, Wang L, Liu J, Bátkai S, et al (2001) Leptin-regulated endocannabinoids are involved in maintaining food intake. Nature 410: 822–825. 11298451 10.1038/35071088

[5] Engeli S, Böhnke J, Feldpausch M, Gorzelniak K, Janke J, et al (2005) Activation of the peripheral endocannabinoid system in human obesity. Diabetes 54: 2838–2843. 16186383 10.2337/diabetes.54.10.2838 PMC2228268

[6] Blüher M, Engeli S, Klöting N, Berndt J, Fasshauer M, et al (2006) Dysregulation of the peripheral and adipose tissue endocannabinoid system in human abdominal obesity. Diabetes 55: 3053–3060. 17065342 10.2337/db06-0812 PMC2228260

[7] Liu J, Zhou L, Xiong K, Godlewski G, Mukhopadhyay B, et al (2012) Hepatic cannabinoid receptor-1 mediates diet-induced insulin resistance via inhibition of insulin signaling and clearance in mice. Gastroenterology 142: 1218–1228. 22307032 10.1053/j.gastro.2012.01.032 PMC3482511

[8] Tam J, Cinar R, Liu J, Godlewski G, Wesley D, et al (2012) Peripheral cannabinoid-1 receptor inverse agonism reduces obesity by reversing leptin resistance. Cell Metab. 16: 167–79. 10.1016/j.cmet.2012.07.002 PMC3832894 22841573

[9] Osei-Hyiaman D, Liu J, Zhou L, Godlewski G, Harvey-White J, et al (2008) Hepatic CB1 receptor is required for development of diet-induced steatosis, dyslipidemia, and insulin and leptin resistance in mice. J Clin Invest 118: 3160–3169. 18677409 10.1172/JCI34827 PMC2491458

[10] Wu J, Kaufman RJ (2006) From acute ER stress to physiological roles of the Unfolded Protein Response. Cell Death Differ 13: 374–384. 16397578 10.1038/sj.cdd.4401840

[11] Zhang K, Shen X, Wu J, Sakaki K, Saunders T, et al (2006) Endoplasmic reticulum stress activates cleavage of CREBH to induce a systemic inflammatory response. Cell 124: 587–599. 16469704 10.1016/j.cell.2005.11.040

[12] Vecchi C, Montosi G, Zhang K, Lamberti I, Duncan SA, et al (2009) ER stress controls iron metabolism through induction of hepcidin. Science 325: 877–880. 19679815 10.1126/science.1176639 PMC2923557

[13] Lee JH, Giannikopoulos P, Duncan SA, Wang J, Johansen CT, et al (2011) The transcription factor cyclic AMP-responsive element-binding protein H regulates triglyceride metabolism. Nat Med. 17: 812–5. 10.1038/nm.2347 PMC3374483 21666694

[14] Zhang C, Wang G, Zheng Z, Maddipati KR, Zhang X, et al (2012) Endoplasmic reticulum-tethered transcription factor cAMP responsive element-binding protein, hepatocyte specific, regulates hepatic lipogenesis, fatty acid oxidation, and lipolysis upon metabolic stress in mice. Hepatology 55: 1070–82. 22095841 10.1002/hep.24783 PMC3319338

[15] Lee MW, Chanda D, Yang J, Oh H, Kim SS, et al (2010) Regulation of hepatic gluconeogenesis by an ER-bound transcription factor, CREBH. Cell Metab 11: 331–339. 20374965 10.1016/j.cmet.2010.02.016

[16] Chanda D, Kim DK, Li T, Kim YH, Koo SH, et al (2011) Cannabinoid receptor type 1 (CB1R) signaling regulates hepatic gluconeogenesis via induction of endoplasmic reticulum-bound transcription factor cAMP-responsive element-binding protein H (CREBH) in primary hepatocytes. J Biol Chem. 286: 27971–9. 10.1074/jbc.M111.224352 PMC3151042 21693703

[17] Chanda D, Kim YH, Kim DK, Lee MW, Lee SY, et al (2012) Activation of cannabinoid receptor type 1 (CB1R) disrupts hepatic insulin receptor signaling via CREBH-mediated induction of Lipin1. J Biol Chem. 287: 38041–9. 10.1074/jbc.M112.377978 PMC3488074 22989885

[18] Chiang JY (2002) Bile acid regulation of gene expression: roles of nuclear hormone receptors. Endocr Rev. 23: 443–463. 10.1210/er.2000-0035 12202460

[19] Hofmann AF (2009) Bile Acids: Trying to Understand Their Chemistry and Biology with the Hope of Helping Patients. Hepatology 49: 1403–1418. 19296471 10.1002/hep.22789

[20] Aranha MM, Cortez-Pinto H, Costa A, da Silva IB, Camilo ME, et al (2008) Bile acid levels are increased in the liver of patients with steatohepatitis. Eur J Gastroenterol Hepatol. 20: 519–525. 10.1097/MEG.0b013e3282f4710a 18467911

[21] Tung BY, Carithers RL Jr (1999) Cholestasis and alcoholic liver disease. Clin Liver Dis. 3: 585–601. 10.1016/s1089-3261(05)70086-6 11291240

[22] Li T, Francl JM, Boehme S, Ochoa A, Zhang Y, et al (2012) Glucose and insulin induction of bile acid synthesis: mechanisms and implication in diabetes and obesity. J Biol Chem. 287: 1861–73. 10.1074/jbc.M111.305789 PMC3265867 22144677

[23] Li T, Matozel M, Boehme S, Kong B, Nilsson LM, et al (2011) Overexpression of cholesterol 7α-hydroxylase promotes hepatic bile acid synthesis and secretion and maintains cholesterol homeostasis. Hepatology 53: 996–1006. 21319191 10.1002/hep.24107 PMC3079544

[24] Nestel PJ, Simons LA, Homma Y (1976) Effects of ethanol on bile acid and cholesterol metabolism. Am J Clin Nutr. 29: 1007–1015. 10.1093/ajcn/29.9.1007 183492

[25] Axelson M, Mörk B, Sjövall J (1991) Ethanol has an acute effect on bile acid biosynthesis in man. FEBS Lett. 281: 155–159. 10.1016/0014-5793(91)80382-d 2015886

[26] Nilsson LM, Sjövall J, Strom S, Bodin K, Nowak G, et al (2007) Ethanol stimulates bile acid formation in primary human hepatocytes. Biochem Biophys Res Commun. 364: 743–747. 10.1016/j.bbrc.2007.10.039 17976534

[27] Song KH, Ellis E, Strom S, Chiang J (2007) Hepatocyte growth factor signaling pathway inhibits cholesterol 7alpha-hydroxylase and bile acid synthesis in human hepatocytes. Hepatology 46: 1993–2002. 17924446 10.1002/hep.21878

[28] Park YJ, Qatanani M, Chua SS, LaRey JL, Johnson SA, et al (2008) Loss of orphan receptor small heterodimer partner sensitizes mice to liver injury from obstructive cholestasis. Hepatology 47: 1578–1586. 18393320 10.1002/hep.22196

[29] Kim I, Ahn SH, Inagaki T, Choi M, Ito S, et al (2007) Differential regulation of bile acid homeostasis by the farnesoid X receptor in liver and intestine. J Lipid Res. 48: 2664–72. 10.1194/jlr.M700330-JLR200 17720959

[30] Jeong WI, Osei-Hyiaman D, Park O, Liu J, Bátkai S, et al (2008) Paracrine activation of hepatic CB1 receptors by stellate cell-derived endocannabinoids mediates alcoholic fatty liver. Cell Metab 7: 227–235. 18316028 10.1016/j.cmet.2007.12.007

[31] Kim DK, Kim YH, Jang HH, Park J, Kim JR, et al (2012) Estrogen-related receptor γ controls hepatic CB1 receptor-mediated CYP2E1 expression and oxidative liver injury by alcohol. Gut 62: 1044–54. 23023167 10.1136/gutjnl-2012-303347 PMC3812689

[32] Waszkiewicz N, Szajda SD, Zalewska A, Konarzewska B, Szulc A, et al (2009) Binge drinking-induced liver injury. Hepatology 50: 1676–1679. 10.1002/hep.23178 19739266

[33] Chen G, Ma C, Bower KA, Shi X, Ke Z, Luo J (2008) Ethanol promotes endoplasmic reticulum stress-induced neuronal death: involvement of oxidative stress. J Neurosci Res. 86: 937–946. 10.1002/jnr.21540 PMC3097119 17941056

[34] Lee AH, Scapa EF, Cohen DE, Glimcher LH (2008) Regulation of hepatic lipogenesis by the transcription factor XBP1. Science 320: 1492–1496. 18556558 10.1126/science.1158042 PMC3620093

[35] Oyadomari S, Harding HP, Zhang Y, Oyadomari M, Ron D (2008) Dephosphorylation of translation initiation factor 2alpha enhances glucose tolerance and attenuates hepatosteatosis in mice. Cell Metab 7: 520–532. 18522833 10.1016/j.cmet.2008.04.011 PMC2474721

[36] Ozcan U, Cao Q, Yilmaz E, Lee AH, Iwakoshi NN, et al (2004) Endoplasmic reticulum stress links obesity, insulin action, and type 2 diabetes. Science 306: 457–461. 15486293 10.1126/science.1103160

[37] Ozcan U, Yilmaz E, Ozcan L, Furuhashi M, Vaillancourt E, et al (2006) Chemical chaperones reduce ER stress and restore glucose homeostasis in a mouse model of type 2 diabetes. Science 313: 1137–1140. 16931765 10.1126/science.1128294 PMC4741373

[38] Rutkowski DT, Wu J, Back SH, Callaghan MU, Ferris SP, et al (2008) UPR pathways combine to prevent hepatic steatosis caused by ER stress-mediated suppression of transcriptional master regulators. Dev Cell. 15: 829–840. 10.1016/j.devcel.2008.10.015 PMC2923556 19081072

[39] Bochkis IM, Rubins NE, White P, Furth EE, Friedman JR, et al (2008) Hepatocyte-specific ablation of Foxa2 alters bile acid homeostasis and results in endoplasmic reticulum stress. Nat Med. 14: 828–836. 10.1038/nm.1853 PMC4095974 18660816

[40] Ozawa K, Miyazaki M, Matsuhisa M, Takano K, Nakatani Y, et al (2005) The Endoplasmic Reticulum Chaperone Improves Insulin Resistance in Type 2 diabetes. Diabetes 54: 657–663. 15734840 10.2337/diabetes.54.3.657

[41] Nakatani Y, Kaneto H, Kawamori D, Yoshiuchi K, Hatazaki M, et al (2005) Involvement of endoplasmic reticulum stress in insulin resistance and diabetes. J Biol Chem. 280: 847–851. 10.1074/jbc.M411860200 15509553

[42] Nguyen P, Leray V, Diez M, Serisier S, Le Bloc’h J, et al (2008) Liver lipid metabolism. J. Anim. Physiol. Anim. Nutr. 92: 272–283. 10.1111/j.1439-0396.2007.00752.x 18477307

[43] Ji C, Mehrian-Shai R, Chan C, Hsu YH, Kaplowitz N (2005) Role of CHOP in hepatic apoptosis in the murine model of intragastric ethanol feeding. Alcohol Clin Exp Res 29: 1496–1503. 16131858 10.1097/01.alc.0000174691.03751.11 PMC1432051

